# Common Risk Factors for Atrial Fibrillation After Transcatheter Aortic Valve Implantation: A Systematic Review from 2009 to 2024

**DOI:** 10.3390/jcdd12030090

**Published:** 2025-02-28

**Authors:** John Fernando Montenegro-Palacios, Sinthia Vidal-Cañas, Nelson Eduardo Murillo-Benítez, Jhon Quintana-Ospina, Carlos Andrés Cardona-Murillo, Yamil Liscano

**Affiliations:** 1 Specialization in Internal Medicine, Department of Health, Universidad Santiago de Cali, Cali 760035, Colombia; sinthia.vidal00@usc.edu.co (S.V.-C.); nemurillob@gmail.com (N.E.M.-B.); jhonquintanaospina@gmail.com (J.Q.-O.); carlos.cardona09@usc.edu.co (C.A.C.-M.); 2 Genetics, Physiology, and Metabolism Research Group (GEFIME), Ciencias de la Salud Universidad Santiago de Cali, Cali 760035, Colombia; 3 Grupo de Investigación en Salud Integral (GISI), Departamento Facultad de Salud, Universidad Santiago de Cali, Cali 760035, Colombia; 4 Department of Research and Education, Clínica de Occidente S.A., Cali 760046, Colombia

**Keywords:** TAVI (transcatheter aortic valve implantation), atrial fibrillation, incidence of atrial fibrillation, post-TAVI complications, cardiac surgical procedures, transcatheter aortic valve replacement, NOAF (new-onset atrial fibrillation)

## Abstract

Transcatheter Aortic Valve Implantation (TAVI) is an effective treatment for severe aortic stenosis in high-risk patients; however, atrial fibrillation (AF) is a common complication associated with the procedure. New-Onset Atrial Fibrillation (NOAF) after TAVI is linked to increased mortality and additional complications. This study aimed to evaluate the incidence of NOAF following TAVI and identify risk factors associated with mortality and the development of thromboembolic events. A systematic review of 18 studies was conducted using databases such as MEDLINE/PubMed, EMBASE, Web of Science, Scopus, Cochrane Library, Google Scholar, Wiley Online Library, SciELO, and Redalyc. No language restrictions were applied, and the search covered studies from 2009 to 2024. The follow-up period ranged from 48 h to 730 days, with a mean of 180 days. Early monitoring and management of AF are essential in patients undergoing TAVI. The incidence of NOAF ranged up to 29.04%, meaning about 29 out of every 100 patients were affected. AF rates varied between 7.2% and 37%, with an average of around 20. Standardizing anticoagulation strategies is important to reduce complications. Randomized studies are needed to evaluate the relationship between AF and post-TAVI mortality and to determine whether AF is a marker of higher risk or an independent factor in these patients.

## 1. Introduction

Transcatheter Aortic Valve Implantation (TAVI) has emerged as an effective treatment alternative for patients with severe aortic stenosis who are considered high risk or ineligible for surgical valve replacement [1,2]. Recent clinical trials have demonstrated the safety and efficacy of TAVI when compared to Surgical Aortic Valve Replacement (SAVR), making it a viable option for a growing number of patients. With advancements in technology and operator experience, the number of TAVI procedures has significantly increased, surpassing 50,000 interventions in the United States, according to the 2016 annual report from the Transcatheter Valve Therapy Registry of the Society of Thoracic Surgeons and the American College of Cardiology [3,4].

TAVI differs inherently from conventional cardiac surgery, particularly in the patient population it serves and the characteristics of the procedure itself [5]. One key difference is the shorter duration of hemodynamic changes during TAVI, which is associated with a notable risk of thromboembolic complications [5,6]. This arrhythmia is especially prevalent in older adults [7].

New-Onset Atrial Fibrillation (NOAF) following TAVI has been linked to poor outcomes, including prolonged hospital stays and an increased risk of in-hospital mortality. Furthermore, NOAF has been associated with a higher likelihood of intrahospital stroke, both in unadjusted and adjusted analyses [8,9]. Given these complications, understanding the risk factors and management strategies for NOAF is essential to improving patient outcomes.

In lower-risk patients, TAVI demonstrated reduced early mortality and composite death or disabling stroke rates, though long-term outcomes were similar [10]. The incidence of stroke after transfemoral TAVI was 2.4%, with prior cerebrovascular events and low glomerular filtration rate as independent predictors: NOAF is associated with stroke with its 6-fold increase in 30-day mortality [11]. Recent studies suggest improved outcomes for TAVI, particularly with transfemoral access and in lower-risk patients [11]. While TAVI appears superior to SAVR in terms of 1-year all-cause mortality, especially for high-risk elderly patients, both procedures show similar rates of postoperative stroke and short-term cardiovascular mortality [12]. Given the increased risk of stroke associated with AF, it is crucial to explore the potential interactions between TAVI and AF both during and after the procedure.

AF is a common complication after TAVI, and its occurrence is associated with worse short- and long-term clinical outcomes [9]. Despite advances in the treatment of aortic stenosis by TAVI, there remains an important gap in the comprehensive understanding of the risk factors predisposing to AF in these patients [13]. Although several studies have explored this relationship, the lack of standardization in definitions and variability in findings limit the creation of effective clinical guidelines for its management. This systematic review, covering studies from 2009 to 2023, aims to analyze and stratify the incidence of post-TAVI AF and its associated risk factors. Consolidating the available evidence is expected to contribute significantly to improving the prevention, early diagnosis, and clinical management of AF and its thromboembolic consequences in patients undergoing TAVI. The results of this review will not only provide a solid basis for the development of more effective policies and guidelines but will also open new opportunities for research on the interactions between AF and other cardiovascular complications in this clinical setting.

## 2. Materials and Methods

### 2.1. Study Protocol

This systematic review was conducted following the guidelines outlined in the Cochrane Collaboration Handbook and reported in accordance with the PRISMA (Preferred Reporting Items for Systematic Reviews and Meta-Analyses) statement [14]. The research was structured using the PICO strategy (Population, Intervention, Comparison, Outcomes) [15]. Prospero register: CRD42024588818.

### 2.2. Research Question

(P): Adult patients undergoing TAVI. (I): Assessment of the incidence of NOAF or paroxysmal AF after TAVI. (C): Patients who do not develop NOAF or AF after TAVI. (O): Incidence of AF, mortality, thromboembolic complications, length of hospital stay, and associated risk factors.

### 2.3. Eligibility Criteria

#### 2.3.1. Inclusion Criteria

- Cohort studies, case–control studies, and clinical trials;
- Manuscripts published between 2009 and 2024;
- Patients of any age and sex who have undergone TAVI (specific valve type and implantation technique must be provided);
- Studies conducted on humans;
- Articles published in Spanish or English;
- Complete data on outcomes and exposures;
- Studies with post-procedural follow-up for a defined period;
- Presence of comorbidities;
- Studies that report association measures (such as risk ratios, odds ratios, or hazard ratios) with corresponding confidence intervals;
- Adequate methodological quality: Studies that meet specific methodological criteria to ensure reliability, including those that account for confounding variables.

#### 2.3.2. Exclusion Criteria

- Systematic reviews, meta-analyses, letters to the editor, commentaries;
- Studies that do not specify TAVI procedure details or patient characteristics;
- Studies without an appropriate comparison group;
- Incomplete data;
- Studies that do not independently analyze the effects of risk factors;
- Pre-print articles;
- Letters to the editor.

### 2.4. Data Sources and Search Strategy

The searches were performed through databases such as MEDLINE/PubMed (via the National Library of Medicine), EMBASE, Web of Science, Scopus, Cochrane Library, Google Scholar, Wiley Online Library, SciELO, and Redalyc. The search covered studies published between 2009 and 2024. The search strategy was developed and executed independently by two researchers (S.M.V. and J.F.M.P) from July 2009 to 2024. Keywords were combined using Boolean operators (AND, OR) (see Appendix A for details). References from relevant articles were reviewed, and additional web searches were conducted to identify any studies not initially captured. Data were managed using Zotero version 6.0 (accessed on 20 August 2024, https://www.zotero.org/).

### 2.5. Study Selection and Data Extraction

Two researchers independently selected potentially eligible studies. All references were imported into the Rayyan QCRI software 1.5.4 (Qatar Computing Research Institute, Ar-Rayyan, Qatar) last updated Rayyan on 1 April 2024. To eliminate duplicates. Initial screening was performed based on titles and abstracts, followed by full-text reviews. Discrepancies were resolved through discussion, and inclusion in the review was decided by consensus.

The two reviewers (S.M.V. and J.F.M.) extracted key information from each primary study, including the following: first author, year of publication, valve type, implantation technique, incidence of AF, time until AF develops, thromboembolic complications, in-hospital mortality, length of hospital stay in days, follow-up in months, and evaluation of surgical mortality risk using the EuroSCOREII through hazard ratios (HRs), confidence intervals (CIs), fold change, *p*-values, and study conclusions. A third reviewer (Y.L.) verified the accuracy and integrity of the recorded data.

### 2.6. Risk of Bias Assessment

The risk of bias in the included studies was assessed by independent reviewers using standardized instruments suitable for cohort studies, case–control studies, and observational designs. Data from this assessment were entered into Review Manager version 5.4^®^ (RevMan).

In this systematic review, the ROBINS-E tool was used to assess the risk of bias in study results across each domain. In addition, the Newcastle–Ottawa scale was used to assess the quality of the included observational studies. Any disagreements that arose during the bias assessment process were addressed and resolved through discussions among the reviewers until a consensus was reached [16,17].

### 2.7. Ethical Considerations

No interventions were made regarding the demographic or physiological variables of the participants in this review. Therefore, this research was considered minimal risk according to Resolution No. 8430 of 1993 of Colombian legislation and the Declaration of Helsinki.

## 3. Results

### 3.1. Characteristics of the Included Studies

**Figure 1:**
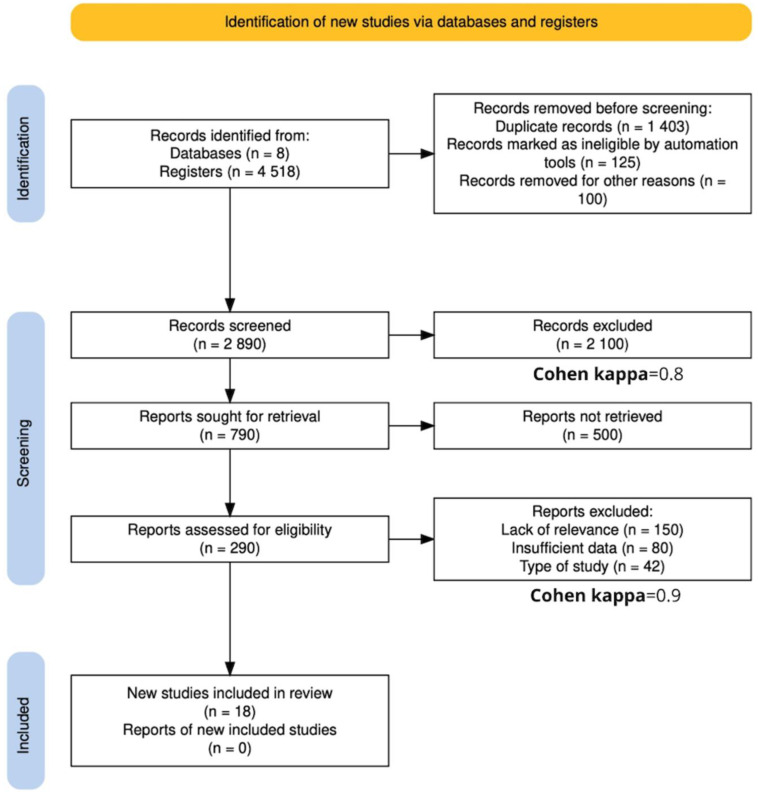
PRISMA flow diagram showing the search and study selection strategy. Cohen’s kappas of 0.8 and 0.9 indicate a high level of agreement between reviewers, lending reliability and validity to the results.

After conducting a comprehensive search across eight databases, a total of 4518 articles were retrieved. Of these, 1403 were identified as duplicates and subsequently removed. The remaining articles underwent a selection process based on their titles and abstracts, resulting in the evaluation of 2890 articles. During this stage, 2100 articles were excluded, and a Cohen’s kappa coefficient of 0.80 was calculated, indicating a high level of agreement between reviewers [18,19,20,21]. A further full-text evaluation of the remaining 790 articles was performed to determine their eligibility for inclusion in the review. Of these, 500 were excluded, and reports excluded were due to lack of relevance (31), insufficient data (56), duplication (9), and type of study (14). A Cohen’s kappa of 0.9 was obtained at this stage, reflecting substantial agreement, though slightly lower than in the initial selection. In the end, 18 studies met all inclusion criteria and were included in the systematic review. The study selection process is detailed in the PRISMA [22] flow diagram shown in Figure 1.

### 3.2. Findings of the Studies

The findings of the studies are explained in different points below.

#### 3.2.1. Incidence of Atrial Fibrillation

Table 1 summarizes the characteristics of the 18 studies reviewed, including cohort, observational, and case–control studies and clinical trials that examined complications in patients undergoing TAVI, particularly AF. The studies report various types of AF [5,6,7,8], including NOAF, preoperative AF, and, in some cases, paroxysmal AF [23,24,25,26,27,28,29,30,31,32,33,34]. The patients were evaluated using the EuroSCORE scale, which is commonly employed to estimate mortality risk in cardiac surgery patients [13]. The average score was 16.37. The most frequently used valves were Edwards SAPIEN (78.5%), SAPIEN 3 (52.7%), and CoreValve (21.5%). The patient population was composed of 48.2% men and 51.8% women, with a mean age of 80.8 years. The most common comorbidities were hypertension, diabetes mellitus, chronic kidney disease, chronic pulmonary disease, and peripheral vascular disease. The follow-up period ranged from 48 h to 730 days, with a mean of 180 days. The overall incidence of paroxysmal or NOAF across studies was 29.04%.

Many of the studies focused on the development of AF within the first two days post-TAVI, as observed in studies such as those by Biviano et al., 2015 [35], Filardo et al., 2010 [24], and Amat et al., 2012 [1], which reported the occurrence of paroxysmal or NOAF within the first 48 h. For example, Biviano et al., 2015 [35] assessed the impact of AF in 1879 patients with electrocardiograms (ECGs). Among these, 113 patients had normal sinus rhythm (NSR) at baseline but developed AF at discharge, while 470 patients had AF at both baseline and discharge. The study found that patients who transitioned from NSR to AF at discharge had the highest all-cause mortality rates at 30 days, showing that AF was a predictor of mortality at one year (adjusted HR = 2.14 for the NSR/AF group and HR = 1.88 for the AF/AF group; *p* < 0.0001 for both groups vs. NSR/NSR).

Similarly, Amat et al., 2012 [1] evaluated 138 consecutive patients with no prior history of AF, finding that AF occurred in 44 patients (31.9%) within a median of 48 h. Predictive factors included left atrial size (odds ratio [OR]: 1.21 per 1 mm/m^2^ increase, 95% confidence interval [CI]: 1.09 to 1.34, *p* = 0.0001) and transapical access (OR: 4.08, 95% CI: 1.35 to 12.31, *p* = 0.019). At 30 days of follow-up, NOAF was associated with a higher rate of stroke or systemic embolism (13.6% vs. 3.2%, *p* = 0.021; adjusted *p* = 0.047 for baseline differences between groups). Additionally, the cumulative incidence of stroke or systemic embolism was 3.2% in the NOAF group vs. 0.023% in those without AF.

#### 3.2.2. Surgical Techniques

Regarding the implantation technique, the transfemoral approach was used most frequently (63.64%), followed by the transapical (38.52%) and transthoracic (22.00%) approaches. For instance, studies by Furuta et al., 2016 [25], Kosmidou et al., 2019 [26], Kompella et al., 2024 [27], and Kalra et al., 2019 [8] reviewed 1959 patients who underwent TAVI and found that older age, major bleeding, and life-threatening bleeding were independent predictors of NOAF. Mortality at 30 days and cumulative mortality at one year were significantly higher in patients with NOAF compared to those without (3.0% vs. 7.4%; *p* = 0.005 and 9.1% vs. 20.8%; *p* < 0.001, respectively).

#### 3.2.3. Mortality Associated with Atrial Fibrillation

Patients with post-TAVI atrial fibrillation (AF) face worse outcomes, as evidenced by increased all-cause mortality, hospital readmissions, and reduced quality of life. Kompella et al., 2024 [27] observed higher one-year all-cause mortality (9.0% vs. 6.1%, *p* = 0.046) and readmission rates (13.1% vs. 8.8%, *p* = 0.014) in patients with AF compared to those without. Furthermore, AF appears to elevate the risk of cardiovascular events, including stroke, heart failure, and bleeding complications, contributing to a heavier disease burden.

Furuta et al., 2016 [25] and Kosmidou et al., 2019 [26] highlighted the prognostic significance of AF following TAVI, linking it to increased cardiovascular mortality and thromboembolic events. Shaul et al., 2015 [7] and Barbash et al., 2014 [5] corroborated these findings, emphasizing the vulnerability of these patients to adverse events. Shahim et al., 2021 [29] and Lee et al., 2023 [30] extended these observations, reporting an association between post-TAVI AF and diminished functional recovery and health-related quality of life.

Kalra et al., 2019 [8] provided further insight into the mechanistic underpinnings, suggesting that inflammation, left atrial remodeling, and prothrombotic states may amplify the risk in this cohort. This growing body of evidence underscores the importance of targeted strategies to mitigate risks in post-TAVI AF patients, including early rhythm control, anticoagulation optimization, and multidisciplinary follow-up.

### 3.3. Risk of Bias Assessment

Using a risk of bias graph created with RevMan 5.4^®^, the assessment of bias risk for the included studies is detailed as follows. Most studies, including those by Furuta et al., 2016 [25], Kosmidou et al., 2019 [26] Shaul et al., 2015 [7], Kompella et al., 2024 [27], Kalra et al., 2019 [8], Shahim et al., 2021 [29], Lee et al., 2023 [30], Barbash et al., 2014 [5], and others, showed a low risk of selection bias, suggesting thorough and well-documented randomization procedures.

Allocation concealment was generally rated as low risk, indicating that the assignment process was sufficiently concealed to minimize selection bias in most studies. However, some studies, like Amat et al., 2012 [1], were rated as having unclear risk, highlighting the need for more explicit documentation or reporting.

Blinding of participants and personnel (performance bias) was rated as high risk in several studies due to the open-label nature of many clinical trials, which could introduce performance bias. For example, the studies by Amat et al., 2012 [1], Biviano et al., 2015 [35], and Filardo et al., 2010 [24] raised concerns regarding the lack of blinding, which could influence both treatment adherence and outcome reporting.

**Figure 2:**
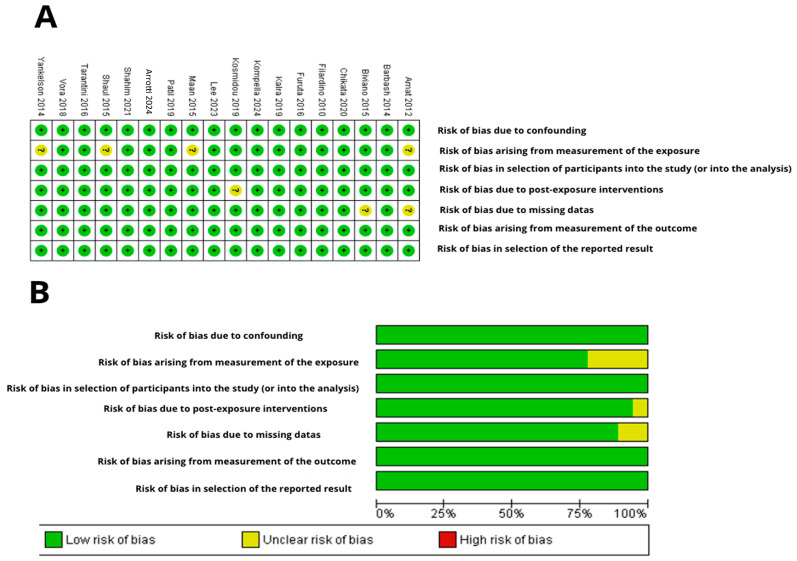
Risk of bias assessment for non-randomized studies using ROBINS-E. (**A**) Summary of risk of bias: the authors’ judgments on each risk of bias item for each included study. The symbol “+” indicates a low risk of bias, the symbol “?” indicates an uncertain risk of bias, and the symbol “-” indicates a high risk of bias [1,5,6,7,8,13,24,25,26,27,28,29,30,31,32,33,34,35]. The colors used are green for low risk of bias, yellow for uncertain risk, and red for high risk of bias. (**B**) Risk of bias graph: authors’ judgments on each risk of bias item presented as percentages across all included studies. Figure created with RevMan 5.

Blinding of outcome assessors (detection bias) was mostly rated as low risk, reflecting the likelihood that outcome assessors were blinded to the intervention groups, as many trials used double or triple blinding to reduce detection bias. However, the handling of incomplete outcome data (attrition bias) showed varying levels of risk across studies (low, unclear, and high). A low risk indicated transparent reporting of participant dropouts and appropriate management of missing data, while unclear or high risks suggested insufficient transparency, which could undermine the validity of the results (See Figure 2).

Selective reporting (reporting bias) varied across studies, with most being rated as low risk, implying that all predefined outcomes were likely reported and study protocols were recorded. However, a few studies had an unclear risk due to insufficient information regarding the reporting of all expected outcomes.

Overall, most studies demonstrated a low risk of bias across key domains, indicating high methodological quality. However, variability in incomplete outcome data and selective reporting warrants careful interpretation of the findings.

Assessment of the risk of bias in the reviewed studies is a fundamental aspect of conducting. Assessment of the risk of bias in the reviewed studies is a fundamental aspect of conducting an assessment (selection, comparability, and outcome).

The NOS can be interchanged with the commonly systematic review. Bias risk assessment in the reviewed studies was examined using the Newcastle Agency for Healthcare Research and Quality Standard Assessment (ARHQ). The more stars, NOS [25]. The NOS scale can only be applied to original studies; therefore, only the lesser the risk of bias in the studies is included. Each study is rated as poor (0–4 *), fair (5–6 *), or good original articles were graded using the NOS scale. The Newcastle–Ottawa scale is a tool for quality (7–9 *). The results from Table 2 indicate that most of the studies included in this review were rated as “good”, suggesting a low risk of bias.

## 4. Discussion

### 4.1. Incidence of Post-TAVI AF

This study conducted a systematic review to evaluate the incidence of NOAF or Paroxysmal Atrial Fibrillation (PAF) following TAVI, as well as the associated risk factors. We found that the incidence of NOAF can reach up to (29.04%), affecting approximately (29 out of every 100) patients. This variability stands out among different patient characteristics, such as advanced age and comorbidities, as well as procedural and surgical technique differences. On the other hand, the study by Ryan et al. (2022) [9] reported an incidence of (9.9%), suggesting that (1 in every 10) patients develops NOAF after TAVR. Likewise, the average time to develop AF was observed. Additionally, the review identified risk factors, such as the presence of comorbidities, which increase the likelihood of developing NOAF. Adverse outcomes associated with NOAF, such as mortality, stroke, and major bleeding, were also quantified. Similarly, we evaluated differences in incidence rates across studies and explored possible causes of variability. Finally, postoperative management strategies, such as adequate monitoring of NOAF, were proposed to reduce complications in patients undergoing TAVR.

On the other hand, Tarantini et al. (2016) [33] reported a higher incidence of AF, reaching up to 35.6%. This variability in the incidence of AF can be attributed to multiple factors. According to the SOURCE XT registry analysis, factors such as the use of a non-femoral access route and balloon post-dilatation were identified as independent predictors of the NOAF. Additionally, differences in patient characteristics, AF detection methods, and the definitions used to classify AF episodes impact the results.

Recent studies, such as that of Arrotti et al. 2024 [13], found an AF incidence of 49.7%, while Patil et al. (2019) [6] reported an even higher incidence of 55%. These differences may be due to heterogeneity in detection methods, definitions used, and patient characteristics, such as advanced age, hypertension, and a history of aortic stenosis. Moreover, the surgical approaches used (e.g., transfemoral vs. transapical access) also influence incidence rates. These results reflect variability in NOAF rates across studies, likely related to patient characteristics or the methodologies employed.

In our systematic review, the average time to develop AF after TAVI was evaluated, finding that it can appear up to 180 days post-procedure. However, studies such as those by Furuta et al., 2016 [25] and Yankelson et al., 2014 [7] reported an average development time of 365 days, while Shaul et al., 2015 [7] found an average of 730 days. In other cases, such as in the same study by Shaul et al., 2015 [7], the development of AF occurred in just 7 days, and in studies by Biviano et al., 2015 [35] and Filardo et al., 2010 [24], it was observed within 2 days. The average incidence of AF in these studies was approximately 30.28%, indicating that NOAF tends to occur more frequently within the first 7 days.

Likewise, it was found that the most commonly used valve is the Edwards SAPIEN and its versions (SAPIEN 3 and SAPIEN XT), present in 78.5% of procedures. The most common surgical technique was transfemoral access, with an average usage of 74.05%, while transapical access was associated with a significantly higher rate of NOAF. Regarding the variability of valves used, studies such as Ryan et al., 2022 [9] reported a NOAF incidence of 9.9% with the SAPIEN valve, while Tarantini et al., 2016 [33] recorded 35.6% and Arrotti et al., 2024 [13] 49.7% with expandable valves. On the other hand, the use of the CoreValve and its versions (Evolut R, PRO, PRO Plus) showed a NOAF incidence of 38% in the study by Shaul et al., 2015 [7] and 20% in that of Barbash et al., 2014 [5], which also reported higher mortality in patients with AF.

AF detection methods included continuous electrocardiographic monitoring during hospitalization, as was the case in the studies by Barbash et al., 2014 [5], Furuta et al., 2016 [25], and Kalra et al., 2019 [8], who also used retrospective analyses of hospital records to detect AF episodes both during hospitalization and in follow-up. This could be related to differences in patient characteristics and methodologies used in the studies.

### 4.2. Associated Risk Factors

The heterogeneity observed in the studies includes variations in implantation techniques, types of valves used, and AF detection methods. Surgical approaches, such as transfemoral and transapical, influence NOAF rates, with higher rates associated with transapical access (Barbash et al., 2014 [5]; Shaul et al., 2015 [7]). Additionally, the choice of valve, such as Edwards SAPIEN or CoreValve, shows differences in AF incidences across studies (Ryan et al., 2022 [9], Arrotti et al., 2024 [13]).

This variability affects the interpretation of results, as AF rates change depending on the combination of techniques and devices used. Studies with the SAPIEN valve report lower rates of AF in comparison to CoreValve (Shaul et al., 2015 [28]; Barbash et al., 2014 [5]). Additionally, detection methods, ranging from continuous ECG monitoring to retrospective analyses, can influence reported rates, making comparison difficult and affecting general conclusions regarding AF incidence post-TAVI (Furuta et al., 2016 [25]; Kalra et al., 2019 [8]).

The most frequent comorbidities in patients with AF following transcatheter aortic valve replacement (TAVR) include pulmonary hypertension in 33.6% of patients, severe renal impairment (with glomerular filtration rate <30 mL/min or on dialysis) in 31.3%, moderate to severe mitral regurgitation in 27.3%, and chronic obstructive pulmonary disease (COPD) in 22.8%. In studies such as that by Tarantini et al. (2016) [33], the main comorbidities associated with AF in patients undergoing TAVI were pulmonary hypertension (33.6%), severe renal impairment (31.3%), moderate to severe mitral regurgitation (27.3%), and COPD (22.8%). On the other hand, in the study by Furuta et al. 2016 [25], the most common comorbidities were hypertension (70.2%), diabetes mellitus (24.2%), COPD (25%), and chronic renal impairment (8.1%). Comorbidities in patients with AF who have undergone TAVR play a significant role in the risk of complications and affect the prognosis. Factors such as pulmonary hypertension, severe renal impairment, moderate to severe mitral regurgitation, and COPD should be considered in clinical evaluation and management, as they significantly increase the risk of AF and the postoperative clinical impact in these patients [25,33].

Other predictive factors for NOAF have been identified, such as left atrial size [OR]: 1.21 for each increase of 1 mm^2^, as reported by Amat et al. 2012 [1]. Non-transfemoral access [HR: 3] and post-balloon dilation [OR: 1.6] were also associated with higher AF rates, particularly in transapical TAVR procedures, where AF rates ranged from 6% to 38%. This is supported by the study by Tarantini et al. 2016 [33], which found an odds ratio of 4.08 for transapical access associated with NOAF. Given these findings, it is essential to carefully select patients for TAVI. It has been suggested that the cardiac team (composed of clinical cardiologists and surgeons) should follow European valve guidelines, establishing criteria such as an aortic valve area <1 cm^2^ or an indexed area <0.6 cm^2^/m^2^ with a mean gradient >40 mm Hg. Pre-procedure planning, including computed tomography angiography of the aorta with multidetector images and echocardiography, should also be performed to assess vascular anatomy and determine the best access route for TAVI.

### 4.3. Clinical Implications and Management

The clinical implications of AF following TAVR are significant, particularly regarding stroke and thromboembolic events, which are more frequent in patients without immediate anticoagulation after the procedure. However, it remains unclear whether antiplatelet therapy should be added to anticoagulation [36]. Experiences with aortic bioprostheses suggest that anticoagulation alone may be sufficient for thromboembolism prevention, although the minimum duration of anticoagulation for TAVI remains uncertain.

Additionally, optimizing antiplatelet therapy is crucial. Dual antiplatelet therapy (DAPT) with aspirin and clopidogrel is commonly used to reduce complications. For patients requiring formal anticoagulation, combinations such as clopidogrel plus warfarin are preferable indefinitely or at least as long as the indication persists, as demonstrated in the study by Vora et al. 2018 [32]. It was found that up to 28.9% of patients with new AF were discharged with oral anticoagulation. However, evidence on antithrombotic management in TAVI patients is limited, leading to variability among centers and clinicians. Recommendations often include lifelong aspirin (75–100 mg/day) and clopidogrel for 3 to 6 months post-TAVI, although randomized data supporting these recommendations are lacking. Recent studies suggest that adding clopidogrel to aspirin may increase bleeding risk without a net ischemic benefit [36].

The study by Vavuranakis et al. 2018 [37] highlights that antithrombotic therapy in patients with AF is complex and recommends assessing both thrombotic risk (CHA_2_DS_2_-VASc) and bleeding risk (HAS-BLED), taking into account patient frailty. Thus, in patients with high thrombotic risk (CHA_2_DS_2_-VASc ≥ 2), combining oral anticoagulants (OACs) with aspirin during the first 3 months is suggested, while antiplatelet therapy alone is preferred in low-risk patients (CHA_2_DS_2_-VASc < 2). However, the ACC/AHA guidelines recommend aspirin monotherapy after TAVR, reserving dual therapy with clopidogrel only for specific cases (recommendation 2b, evidence BR) (Granger et al., 2022 [38]). Additionally, the STS/ACC registry, which included 21,131 patients, showed that those treated with direct oral anticoagulants (DOACs) had lower mortality and bleeding rates compared to those treated with vitamin K antagonists (VKAs), with a mortality of 15.8% versus 18.2% (adjusted HR 0.92; *p* = 0.043) and a reduction in bleeding risk (HR 0.81; *p* < 0.001). On the other hand, the 4D-CT substudy of the ATLANTIS trial found that apixaban reduced valvular thrombosis at 3 months compared to antiplatelet therapy (8.9% vs. 15.9%; *p* = 0.011). Finally, the Popular TAVI trial by Nijenhuis et al., 2020 [39] concluded that in post-TAVR patients treated with OAC, the incidence of major bleeding was lower with OAC alone compared to OAC plus clopidogrel (21.7% vs. 34.6%; RR 0.63; *p* = 0.01), suggesting opportunities for future research on optimal antithrombotic therapy in this population [37,39].

Information bias is another recurring issue. Kompella et al. 2024 [27] recorded comorbidities and complications heterogeneously, affecting the accuracy of the analysis. Barbash et al. 2014 [5] also presented information bias due to a lack of clarity in follow-up duration and long-term mortality outcomes.

Several therapeutic alternatives have been identified to optimize outcomes in diabetic patients with severe aortic stenosis and ventricular dysfunction undergoing TAVI. Among these, the impact of SGLT2 inhibitors on cardiac remodeling and long-term clinical outcomes stands out. A recent shift in understanding aortic stenosis has led to its recognition not only as an isolated valvular disease but also as a condition affecting the myocardium. This paradigm shift has driven the search for therapeutic strategies that address both the valvular pathology and promote left ventricular recovery. In this context, studies have demonstrated that SGLT2 inhibitors offer multiple benefits in patients undergoing TAVI. For instance, patients treated with these medications experienced a higher rate of functional recovery of the left ventricle, particularly those with an initial ejection fraction ≤ 30%. Furthermore, over a median follow-up of 24 months, the use of SGLT2 inhibitors was associated with a significant reduction in the incidence of major adverse cardiovascular events, all-cause mortality, and hospitalization for heart failure compared to those who did not receive this therapy [40]. The potential of SGLT2 inhibitors to reduce cardiac fibrosis and improve microvascular function, as demonstrated in preclinical models, could significantly support structural and functional recovery of the left ventricle after TAVI. This opens new possibilities for therapeutic intervention targeting the myocardium, addressing an unmet clinical need. Although medical therapy alone has not shown efficacy in slowing the progression of aortic stenosis, its combination with TAVI could play a key role in cardiac remodeling and long-term prognosis. The implementation of strategies that integrate TAVI with the use of SGLT2 inhibitors could transform the therapeutic approach, significantly improving clinical outcomes [41,42,43]. SGLT2 inhibitors emerge as a promising option for diabetic patients with severe aortic stenosis and cardiac damage undergoing TAVI, representing a new perspective in optimizing clinical management for this population [40].

Finally, confounding bias is observed in studies like that of Kalra et al. 2019 [8], where multiple comorbidities in patients with AF, such as renal insufficiency, chronic lung disease, and heart failure, are not adequately controlled. These conditions increase both the incidence and complications of AF post-TAVI, which negatively affects clinical outcomes and complicates the interpretation of findings. To improve the accuracy of future studies, it would be essential to adjust analyses based on these comorbidities and consider their management in postoperative treatment. Additionally, incorporating preventive strategies would help reduce the risk of adverse events, providing more valid and generalizable conclusions on AF outcomes post-TAVI. Without these adjustments, biases limit the ability to generalize findings and affect the internal validity of the studies [8,26].

The assessment of the strength of clinical recommendations in the reviewed studies is based on the quality of the evidence provided, including both cohort and observational studies. Most studies, such as Furuta et al. 2016 [25] and Shaul et al. 2015 [28], present a low risk of selection bias due to the use of well-defined cohorts and adequate randomization in some cases. However, studies like Kalra et al. 2019 [8] present a higher risk of confounding bias, as not all comorbidities were adequately controlled in the analyses, which may affect the strength of the conclusions.

To assess the risk of bias, tools such as the Newcastle–Ottawa scale, which allows for the rating of the quality of observational studies, and the RevMan software version 5.4^®^, which facilitated the identification and charting of bias risk in each of the included studies, were used. Kompella et al. 2024 [27], for example, stood out for its low risk of bias in most of the evaluated categories, which strengthens the clinical recommendations derived from this study.

However, studies like Barbash et al. 2014 [5] presented a higher risk of information bias due to a lack of clarity in long-term follow-up and in the documentation of clinical outcomes, which affects the strength of their recommendations

### 4.4. Study Limitations

The systematic review must consider various biases and limitations present in the analyzed studies. The study by Chikata et al. 2020 [31] is based on a TAVI registry with a relatively limited number of institutions and patients, which restricts the generalization of the findings. Additionally, its retrospective analysis carries the risk of confounding due to unaccounted factors, as some data may not have been adequately recorded. Temporal variability in the prognostic impacts of gender and AF is another relevant aspect, as recent studies suggest changes in survival according to the intervention period. Likewise, the disproportionate gender representation, with a higher number of female patients, may influence the results and their applicability to broader populations. On the other hand, Furuta et al. 2016 [25] highlighted that, as a non-randomized registry, there are differences in baseline clinical parameters that may affect the results. The detection of NOAF was conducted through continuous monitoring limited to the hospital stay, which may underestimate the actual incidence of NOAF by failing to capture episodes after discharge. Additionally, there is a risk of overestimation, as NOAF was defined as an episode of 30 s or longer, potentially omitting more prolonged and clinically relevant episodes. The lack of data on the duration and characteristics of AF, as well as on pharmacological management, limits the analysis of the relationship between AF, stroke, and mortality. Additionally, variations in TAVI practices across centers and over the study period may have influenced the results, underscoring the need to address these limitations in future research [25].

The review of 18 studies on post-TAVI AF reveals significant limitations due to heterogeneity among studies, publication bias, and insufficient long-term follow-up data. Variability in implantation techniques and valve types (Edwards SAPIEN vs. CoreValve) makes it difficult to compare results across studies. Additionally, methods for detecting AF vary from continuous monitoring to retrospective reviews, which influences reported incidence rates of post-TAVI AF [26,35].

Publication bias also affects the reliability of the evidence, as some studies report only statistically significant findings, omitting less favorable data. Finally, the lack of extended follow-up limits understanding of the long-term effects of AF on mortality and thromboembolic complications. These limitations highlight the importance of conducting future studies with standardized methodologies and extended follow-up to improve the robustness of clinical recommendations for patients with post-TAVI AF [25,28].

A key aspect of this systematic review is the inclusion of a large number of studies addressing diverse populations of patients undergoing TAVI, which strengthens the external validity of our findings and allows for the generalization of identified risk factors for AF to different subgroups. The diversity of the included populations, varying in terms of age, cardiovascular comorbidities, and demographic characteristics, provides a more comprehensive view of the factors that may influence the development of post-TAVI AF. For example, the study by Tarantini et al. 2016 [33] involved 2706 patients from 99 centers in 17 countries, lending robustness to its results, while the study by Vora et al. 2018 [32] included 13,556 patients, with 1.138 (8.4%) developing NOAF, revealing significant differences based on the type of surgical access: 4.4% in patients with transfemoral access compared to 16.5% in those with non-transfemoral access. Additionally, the study by Shahim 2021 [29], which included 781 patients, identified additional risk factors for postoperative AF, such as the type of procedure (higher incidence in SAVR vs. TAVR), advanced age, NYHA functional class III or IV, non-transfemoral access (hazard ratio of 3), and post-balloon dilation (odds ratio of 1.6). To ensure the validity and reliability of the included studies, a rigorous methodology based on standardized quality assessment tools, such as ROBINS-E [44] and the Cochrane Risk of Bias Tool [45], was used. This ensures that only studies with a high level of methodological quality are included, reinforcing confidence in the review’s results and conclusions, highlighting the rigor of the analysis, and ensuring that the derived recommendations have a strong evidence base.

In the reviewed studies, several types of biases were identified that affect the validity of the results. Selection bias is evident in the study by Furuta et al. 2016 [25], as patients with specific comorbidities (diabetes, hypertension) were included, limiting the representativeness of the general population. Similarly, Shaul et al. 2015 [28] presented a high risk of selection bias in its case–control design due to the potential lack of comparability between groups.

### 4.5. Future Directions and Research

This systematic review makes a significant contribution to the current knowledge on post-TAVI AF by consolidating and analyzing data from multiple studies, focusing on identifying specific risk factors associated with AF, such as advanced age, hypertension, renal insufficiency, and the presence of cardiovascular comorbidities. The integration of data from diverse cohorts and clinical contexts provides a more comprehensive view of the factors that predispose to AF in patients undergoing TAVI, enhancing our understanding of the condition and its implications. Furthermore, by applying a rigorous methodology to assess the quality of the included studies, this review strengthens the validity of its conclusions and provides a solid foundation for future research and clinical practice. The identification of these key risk factors and the consideration of individual aspects in treatment reflect a significant advancement in the personalized care of post-TAVI AF patients, thus contributing to progress in the field of interventional cardiology and the improvement of clinical outcomes [25,26,31].

Post-TAVI antithrombotic therapy is increasingly relevant, as transcatheter valve thrombosis is more common than expected, with an incidence of 1–5% in asymptomatic cases and up to 40% in symptomatic ones. Currently, the use of dual antiplatelet therapy is based on coronary approaches, but evidence of its duration and efficacy is lacking. Clinical practice remains empirical and varies across guidelines, with no clear consensus, especially in patients requiring oral anticoagulation. Additionally, “subclinical leaflet thrombosis” is frequent, highlighting the need for trials to determine the optimal strategy to reduce embolic risks in these patients [37,38].

The quality of evidence from the included studies was crucial for validating the reliability of post-TAVI AF findings. Most randomized and cohort studies utilized validated methods for measuring exposures and outcomes. However, performance bias was notably high due to the open nature of many trials, where both participants and researchers were aware of treatment assignments, potentially influencing results. While most studies reported a low risk of bias in outcome assessments, inconsistencies regarding incomplete data and selective reporting suggest a need for more transparent reporting practices and rigorous methodologies [7,24,34,35].

Advances in technology and the high risk of AF, which can occur in 1 in 10 patients undergoing TAVI, have led to adverse outcomes such as mortality, stroke, major bleeding, permanent pacemaker implantation (PPM), and longer hospital stays [25]. However, further studies comparing transfemoral and transapical approaches in controlled settings are needed to reduce outcome variability. Additionally, the anticoagulation strategy following TAVI remains an area requiring further investigation. Many patients undergoing TAVI already require anticoagulation, and the balance between thromboembolic prevention and bleeding risk needs to be carefully considered.

For future studies, it is recommended to investigate whether NOAF after TAVI is merely a risk marker or a causal factor in adverse postoperative outcomes. Current findings show that NOAF is associated with significant complications, such as increased mortality, stroke risk, and bleeding events, but evidence remains insufficient to establish a direct causal relationship. Future randomized studies should focus on standardizing the monitoring and treatment of NOAF in TAVI patients to improve outcomes and clarify its role in these adverse events.

Finally, our systematic review highlights the importance of monitoring and treating AF appropriately in TAVI patients. Randomized studies are needed to establish whether AF is merely a marker of increased risk or an independent risk factor in TAVI patients.

## 5. Conclusions

Identifying key risk factors such as advanced age, enlarged left atrial size, trans apical access, and cardiovascular comorbidities is valuable for understanding the incidence of post-TAVI AF. This information can support healthcare professionals in anticipating complications and adopting a more personalized approach to patient care. Additionally, the findings suggest that early AF monitoring and the careful selection of antithrombotic strategies may help reduce complications and improve outcomes after TAVI.

However, it is important to recognize the limitations of this review, including study heterogeneity, potential biases, and the limited availability of long-term data. These constraints underscore the need for caution when interpreting the results and highlight areas for future research. Addressing these limitations through more rigorous and standardized methodologies will be crucial for refining our understanding of post-TAVI AF and improving patient management strategies.

## Figures and Tables

**Table 1:** Synthesis of studies included in the systematic review.

Author and Year	Study Type	Mean Age (Years)	Sex (%) Male	Valve Type	Implantation Technique	AF Incidence (%)	Time to AF (Days)	Thromboembolic Complications (%)	In-Hospital Mortality (%)	Hospital Stay (Days)	Association Measure and CI	Follow-Up (Months)	EuroSCORE II
Furuta et al., 2016 [25]	Cohort	82 ± 7.5	52.3	CoreValve 21.5%, Edwards SAPIEN 78.5%	Transfemoral (58.4%), Transapical (32.9%)	7.60%	365	0.70%	4.72 days	HR: NOAF was an independent predictor of 30-day and 1-year mortality	HR: 2.16; 95% CI: 1.06–4.41; *p* = 0.033, HR: 2.12; 95% CI: 1.42–3.15; *p* < 0.001	12	21.8 ± 14.3
Kosmidou et al., 2019 [26]	Cohort	65–74	65.4	SAPIEN XT, SAPIEN 3	Transfemoral (78%), Transthoracic (22%)	100%	60	9.30%	26.10%	30 days	RR for OAC + APT: 0.43; 95% CI: 0.22 to 0.85; *p* = 0.015, RR for APT: 0.32; 95% CI: 0.16 to 0.65; *p* = 0.002	60	Not specified
Shaul et al., 2015 [28]	Case–control	82 ± 6.4	46	CoreValve 80%	Transfemoral (84%)	RS 66%, Paroxysmal AF 18%, Non-paroxysmal AF 16%	730	7.50%	38%	7 days	HR = 2.76; 95% CI: 1.63–4.66; *p* < 0.001	24	16.6
Kompella et al., 2024 [27]	Cohort	82 ± 7.5	58	Sapien 3.7%, Sapien XT 4.3%, Sapien 3 52.7%, Evolut R 11.9%, Evolut PRO 7.1%, Evolut PRO Plus 20.2%	Transfemoral (89.7%), Transapical (1.8%)	Preoperative AF 50.6%, Paroxysmal AF 49.4%	7	1.20%	5%	2.6 ± 3.5	RR: 1.55; 95% CI: 1.17 to 2.06; *p* = 0.003	52	Not specified
Kalra et al., 2019 [8]	Cohort	80.2	51.6	Not specified	Transfemoral (85.8%)	NOAF 14.1%	Not specified	Not specified	2%	Not specified	TAVI OR 1.57; 95% CI: 1.21–2.04, AVR OR: 1.36; 95% CI: 1.08–1.70	Not specified	Not specified
Shahim et al., 2021 [29]	Cohort	74 ± 5	67	Not specified	Not specified	Early 19.5%, Late 7%	Early 365 days, Late > 365 days	Early 6.6%, Late 5.4%	Early 2.2%, Late 5.2%	2.34 ± 1.81	OR 1.04; 95% CI: 0.52–2.08; *p* = 0.90	12	1.5 ± 1
Lee et al., 2023 [30]	Cohort	79.5 ± 6.8	67	SAPIEN XT or 3, CoreValve, Evolut R, Other	Transfemoral	16.40%	Not specified	2%	16.40%	30 days	Welch’s *t*-test evaluated differences	369 days	12.1 ± 9.5
Barbash et al., 2014 [5]	Cohort	84 ± 8	43	SAPIEN (87%), SAPIEN XT (6%), CoreValve (6%)	Transfemoral (74%), Transapical (25%)	20%	Not specified	25%	28.80%	3.5 ± 6.3	Baseline AF: higher 1-year mortality (28.8% vs. 18%, *p* = 0.01)	12 months	27.7
Chikata et al., 2020 [31]	Cohort	Variable	Men without AF: 24.4%, Men with AF: 7%	Not specified	Transfemoral	24.30%	Not specified	Not specified	141 (13.0%), 59 (5.4%), 183 (16.8%)	30 days	IQR based on Shapiro–Wilk test	385 days	4.5
Arrotti et al., 2024 [13]	Cohort	82.2 ± 5.2	49.7	S. expandable, B. expandable	Transfemoral, Non-transfemoral	51%	30 days	Not specified	35.6% and 38.2%	30 days	OR 1.65; 95% CI: 1.15–2.38	24 months	Not specified
Patil et al., 2019 [6]	Cohort	82	55	Not specified	Not specified	57%	6.2 days	1.24%	5.50%	4.9 to 6.2 days	T-tests and chi-square tests	3 years	Not specified
Vora et al., 2018 [32]	Cohort	85	38.6	Not specified	Transfemoral (36.3%), Transapical (43.1%)	8.40%	365	7.20%	30.10%	7.2 days	HR 1.37; 95% CI: 1.19–1.59 for stroke, Adjusted HR 1.50; 95% CI: 1.14–1.98 for stroke, Adjusted HR 1.24; 95% CI: 1.10–1.40 for bleeding	12	Not specified
Yankelson et al., 2014 [7]	Cohort	82.4	35.5	Not specified	Not specified	8.20%	365 days	4.20%	34.90% after one year	10.5 days	Mortality HR: 2.2; 95% CI: 1.3–3.8; *p* = 0.003, HR 1.5; 95% CI: 0.5–4.1; *p* = 0.390	12	21.88
Tarantini et al., 2016 [33]	Cohort	NOAF: 82.8 ± 6.0, Pre-existing AF: 81.6 ± 5.8, RSl: 81.0 ± 6.5	42.8	Not specified	Transapical (55.8%), Transfemoral (39.1%), Transaortic (5.1%)	7.20%	30 days	8.60%	32.10% all causes	10.6 ± 8.1 days	HR for NOAF: 1.96; 95% CI: 1.39–2.76; *p* = 0.0001	24	21.6 ± 12.4
Amat et al., 2012 [1]	Cohort	79 ± 8	54	Not specified	Transfemoral (27.5%), Transapical (72.5%)	31.90%	2 days	13.60%	9.10%	9 days	OR for mortality: NOAF 9.1% vs. no NOAF 6.4%; *p* = 0.57	20	21.7 ± 15.7
Maan et al., 2015 [34]	Case–control	84.18 ± 6.83	47	Edwards SAPIEN	Transapical	15%	6.7 days	3%	27% all causes	11 days	NOAF OR 5.05, 95% CI: 1.40 to 18.20; *p* = 0.013	1 month	14.33 ± 12.24
Biviano et al., 2015 [35]	Prospective Clinical Trial	86.4 (80.5, 90.0)	47.8	Medtronic CoreValves	Transfemoral, Transapical	14.20%	2 days	17.40%	14.20% after one year	9 days ± 7 to 14 days	Mortality at 30 days: HR (*p* < 0.0001 among all groups; 14.2% RS/FA vs. 2.6% RS/RS; HR = 3.41; *p* = 0.0002)	12 months	Not specified
Filardo et al., 2010 [24]	Cohort	74.2 [66.7, 80.1]	63.9	Not specified	Not specified	37%	2 days	Not specified	13%	8.4 days	NOAF POST-AVR associated with higher risk of death (RR: 1.48; 95% CI: 1.12–1.96)	10 years	Not specified

HR: hazard ratio; NOAF: New-Onset Atrial Fibrillation; OAC: oral anticoagulant; APT: antiplatelet therapy; CI: confidence interval; RR: risk ratio; TAVI: Transcatheter Aortic Valve Implantation; OR: odds ratio; POST-AVR: Posterior Transcatheter Aortic Valve Replacement; IQR: interquartile range.

**Table 2:** Newcastle–Ottawa scale quality assessment form for non-randomized studies included in the review.

Study	Selection (4 Score)	Comparability (2 Score)	Result (3 Score)	Total (9 Score)
Shaul et al., 2015 [28]	****	**	***	9
Kompella et al., 2024 [27]	***	**	***	8
Furuta et al., 2016 [25]	***	**	***	8
Kosmidou et al., 2019 [26]	***	**	**	7
Kalra et al., 2019 [8]	***	**	*	6
Shahim et al., 2021 [29]	****	*	**	7
Lee et al., 2023 [30]	***	**	**	7
Barbash et al. 2014 [5]	****	*	***	8
Arrotti et al., 2024 [13]	***	**	***	8
Patil et al., 2019 [6]	****	**	**	8
Vora et al., 2018 [32]	****	**	***	9
Yankelson et al., 2014 [7]	***	**	**	7
Tarantini et al., 2016 [33]	****	**	*	7
Amat et al., 2012 [1]	***	**	*	6
Maan et al., 2015 [34]	****	*	**	8
Biviano et al., 2015 [35]	****	**	*	7
Filardo et al., 2010 [24]	***	**	**	7
Chikata et al., 2020 [31]	****	**	**	8

Description. (*) = poor (0–4 *), fair (5–6 *), or good (7–9 *).

## Data Availability

Data are contained within the article.

## Appendix A

### Appendix A.1

In Table A1, the surgery dates of the studies analyzed in this work are shown. This table demonstrates significant heterogeneity not only in the timing of surgeries but also in sample sizes, which range from as few as 200 to more than 48,000 participants. The time frames span nearly three decades (1997 to 2024), reflecting changes in patient selection criteria, device technology, and operator expertise. Some studies do not specify exact recruitment periods but rather reference larger registries or trials (e.g., PARTNER 3, PARTNER II, SOURCE XT), further complicating direct comparisons. These factors clearly influence the incidence of AF, given the diverse patient populations, distinct procedural approaches, and evolving definitions of new-onset or existing AF.

**Table A1:** Summary of studies investigating atrial fibrillation in patients undergoing Transcatheter Aortic Valve Implantation (TAVI).

Author	Surgery Period	Number of Patients (n)
Yankelson et al., 2014 [7]	September 2008 to April 2013	380
Amat-Santos et al., 2021 [1]	May 2007 to May 2011	138
Tarantini et al., 2016 [33]	Not specified	2706
Vora et al., 2018 [32]	2011 to 2015	13,556
Shahim et al., 2021 [29]	Not specified (PARTNER 3 trial)	781
Chikata et al., 2020 [31]	May 2010 to February 2020	1088
Furuta et al., 2016 [25]	2015 to 2020	1959
Kosmidou et al., 2019 [26]	Not specified (PARTNER II trial period)	1621
Patil et al., 2019 [6]	2012 to 2015	7266
Arrotti et al., 2024 [13]	2012 to 2022	759
Barbash et al., 2020 [5]	2007 to 2013	371
Biviano et al., 2015 [35]	Up to February 2013	1879
Lee et al., 2023 [30]	June 2015 to June 2018	660
Kalra et al., 2019 [8]	January 2012 to September 2015	48,715 (TAVI hospitalizations)
Maan et al., 2015 [34]	June 2008 to October 2012	137
Shaul et al., 2015 [28]	August 2008 to November 2014	319
Filardo et al., 2010 [24]	January 1997 to December 2006	1039
Kompella et al., 2024 [27]	From February 2012 (end date not specified)	1098 with AF (+1787 without AF)

### Appendix A.2

References search algorithms:

(“Atrial Fibrillation” OR “Fibrillation, Atrial” OR “Fibrillations, Atrial” OR “Auricular Fibrillation” OR “Auricular Fibrillations” OR “Fibrillation, Auricular” OR “Fibrillations, Auricular” OR “Persistent Atrial Fibrillation” OR “Atrial Fibrillation, Persistent” OR “Atrial Fibrillations, Persistent” OR “Fibrillation, Persistent Atrial” OR “Fibrillations, Persistent Atrial” OR “Persistent Atrial Fibrillations” OR “Familial Atrial Fibrillation” OR “Atrial Fibrillation, Familial” OR “Atrial Fibrillations, Familial” OR “Familial Atrial Fibrillations” OR “Fibrillation, Familial Atrial” OR “Fibrillations, Familial Atrial” OR “Paroxysmal Atrial Fibrillation” OR “Atrial Fibrillation, Paroxysmal” OR “Atrial Fibrillations, Paroxysmal” OR “Fibrillation, Paroxysmal Atrial” OR “Fibrillations, Paroxysmal Atrial” OR “Paroxysmal Atrial Fibrillations”) AND (“Transcatheter Aortic Valve Replacement” OR “Transcatheter Aortic Valve Implantation” OR “TAVI”) AND (“incidence”) AND (“Risk Factors” OR “Risk Assessment”
